# BCL-3 Promotes Intracerebral Hemorrhage Progression by Increasing Blood–Brain Barrier Permeability, Inflammation, and Cell Apoptosis via Endoplasmic Reticulum Stress

**DOI:** 10.1155/2023/1420367

**Published:** 2023-09-13

**Authors:** Hao Yin, Zhongying Ran, Tao Luo, Zexin Jin, Jun Ma

**Affiliations:** Department of Neurosurgery, Guizhou Provincial People's Hospital, China

## Abstract

**Background:**

Intracerebral hemorrhage (ICH) is among the common types of stroke with high mortality and morbidity. Molecular biomarker selection is crucial for ICH diagnosis and treatment. However, the identification of ICH-related biomarkers remains inadequate.

**Materials and Methods:**

*In vivo* and *in vitro* ICH models were generated and transfected with silenced B-cell lymphoma-3 (BCL-3 and siRNA BCL-3), overexpressed BCL-3, and endoplasmic reticulum stress (ERS) agonist (2-CLHA). Hematoxylin–eosin staining and transmission electron microscopy were used to observe the transfected cells. RNA sequencing was performed *in vivo* on the sham and ICH groups. The blood–brain barrier (BBB) permeability was evaluated by determining Evans blue dye extravasation, transendothelial electrical resistance, and paracellular permeability. Moreover, tight junction-, cell apoptosis-, and endoplasmic reticulum stress- (ERS-) related proteins were evaluated through real-time quantitative PCR, western blotting, immunohistochemistry, and TUNEL staining. The levels of inflammatory cytokines were measured through the enzyme-linked immunosorbent assay.

**Results:**

RNA-seq revealed that BCL-3 acts as a key player. BCL-3 promotes ICH progression by increasing BBB permeability, ERS, inflammation, and cell apoptosis. Silencing of BCL-3 slows ICH progression by reducing BBB permeability and inflammation and terminating cell apoptosis and ERS *in vitro* and *in vivo*.

**Conclusion:**

Our study identified ICH biomarkers and elucidated the role of BCL-3 in ICH for the first time.

## 1. Introduction

Intracerebral hemorrhage (ICH) is among the most prominent type of stroke and accounts for 10%–15% of all strokes. It is associated with high mortality and morbidity [1, 2]. ICH patients exhibit a mortality rate close to 40% in 1 month, and survivors usually develop legacy paralysis, aphasia, and some severe disabilities. Unfortunately, only a few proven effective acute or preventive treatments are available for ICH. ICH patients typically develop primary brain injury due to mechanical damage and hematoma mass formation in brain tissues [3]. Some ICH patients with secondary brain injury exhibit nerve function damage. [4, 5]. ICH patients also exhibit some pathological changes that occur in brain tissues around the hematoma, including blood–brain barrier (BBB) damage, cerebral edema, and cell death [6]. Understanding the mechanisms of inflammatory response, amino acid toxicity, proteolytic enzyme expression, and the toxic effects of products released from the hematoma can help elucidate these pathological changes [5, 6]. Among them, cell death is an essential factor in secondary brain injury after ICH.

How to effectively predict ICH occurrence has become a recent research hotspot, and molecular markers facilitate comprehension of the pathogenesis, targeted therapy, and prognosis of ICH [7]. Mounting studies have demonstrated that RNA biomarkers, such as miRNA, lncRNA, and mRNAs, play essential roles in the prediction, diagnosis, treatment, and prognosis of ICH [8–11]. Developed RNA sequencing (RNA-seq) technologies have allowed the early and accurate identification of biomarkers. For example, Cheng et al. [11] revealed that differentially expressed miRNA, their putative mRNA targets, and associated pathways might offer diagnostic biomarkers and indicate therapeutic targets for ICH treatments in humans. Global transcriptome profiling performed using RNA-seq revealed that differentially expressed genes (DEGs) related to inflammation and immune responses were highly expressed in ICH patients [12]. While most biomarkers were identified, very few were verified.

We first investigated biomarkers using RNA-seq. Then, DEGs that might have essential roles in ICH were further verified. *In vivo* and *in vitro* analyses were performed to probe into the role of these DEGs in ICH and clarify how they regulate ICH progression.

## 2. Material and Methods

### 2.1. ICH In Vivo Model Construction

In total, 56 male rats (8–9 weeks, 180–200 g) were obtained and housed in humidity and temperature-controlled environments on a 12 h : 12 h light–dark cycle with free access to water and food. Rats were randomly divided into three groups (sham, ICH-24 h, and ICH-72 h) and four groups (sham, ICH, ICH+NC, and ICH+BCL-3^kd^), with 8 rats per group.

The *in vivo* ICH model was constructed using the ICH method described by Kung et al. [13] with slight modifications. Briefly, the ICH model was established using collagenase type IV. The rats were fasted for 12 h before the operation, with water consumption stopped 4 hours before the operation. The animals were anesthetized with intraperitoneal administration of 0.4% sodium pentobarbital. A T-shaped incision was made on the scalp, a 3 × 1 × 5 mm injection point was created on the skull, the skull was drilled, the solution was slowly injected by using stereotaxic apparatus, and the needle was retained for 2 min. The needle was slowly withdrawn, and the skull was sealed. The skin was then sutured. The rats were housed in a ventilated room after the operation. The mice in the ICH group were injected with 2.0 *μ*L collagenase (4 U)/heparin (2 U in 1 *μ*L sterile normal saline), and those in the control group were injected with 2.0 *μ*L normal saline by using stereotaxic apparatus (bregma [3.5 mm, 0.2 mm], depth [5.5 mm]). The animal experiments were approved by the Ethic Committee of Guizhou Provincial People's Hospital.

### 2.2. In Vitro Model Construction and Transfections

BMECs were obtained from the rats. Briefly, the rats were sacrificed, and their brains were immersed in 75% ethanol to preserve the cerebral cortex. After rinsing the cerebral cortex three times with the D-Hanks solution, the cortex was placed in DMEM. Then, the cortex was cut into pieces, and 0.1% collagenase type II (containing 30 U/mL DNase I, 1 mL/brain) was added. After homogenization, the pieces were digested in a water bath at 37°C for 1.5 h. Then, the mixture was treated with 20% bovine serum albumin and centrifuged at 1000 g for 20 min. The sediment obtained through centrifugation was retained, supplemented with 0.1% collagenase/dispase (containing 20 U/mL DNase I), digested in the water bath at 37°C for 1 h, and centrifuged at 1000 g for 20 min. Finally, the precipitate was washed with DMEM and cultured with 20% fetal bovine serum and 100 *μ*g/mL heparin sodium at 37°C under 5% CO_2_.

For *in vitro* ICH model construction, BMECs were treated with iron hemoglobin at a concentration of 10 *μ*M [14] (ICH group) and blank control (blank). Then, the ICH cells were transfected with negative control (ICH+NC), silencing RNA BCL-3 (ICH+siRNA BCL-3), vector (ICH+vector), BCL-3 overexpression plasmid (ICH+BCL-3), and an agonist 2-C1HA (ICH+siRNA BCL-3+2-C1HA) by using Lipofectamine^®^2000 (Invitrogen; Thermo Fisher Scientific, Inc.) at 37°C for 2 days. RNA shRNA and the plasmid were obtained from GenePharma Co., Ltd. (Shanghai, China).

### 2.3. Evans Blue Dye Extravasation

Destruction of the BBB in the brain tissue can increase capillary permeability. Albumin bound to Evans blue dye (EBD) can enter the brain tissue through the BBB, thereby staining the brain tissue. Thus, BBB permeability can be determined. Before the EBD extravasation experiment, 2% EBD (2 mL/kg; Sigma, USA) was injected into the rat tail vein 30 min before the animals were sacrificed. After the rats were anesthetized with 1% sodium pentobarbital (30–40 mg/kg), their chest cavity was opened, and 200–300 mL of heparin saline (0.9% sodium chloride +20 U/mL heparin sodium) was intracardially perfused. The brain was decapitated for sagittal cutting to separate the hippocampus. Part of the brain tissue was frozen and sectioned immediately. The remaining part was weighed, placed in dimethylformamide for 60 h at 60°C, and centrifuged at 1000 rpm for 5 min. The absorbance of the supernatant obtained at 620 nm was measured using a spectrophotometer. Data analysis was performed using Origin software (v7.0), and the EBD content was calculated from the previously plotted standard curve.

### 2.4. Hematoxylin–Eosin Staining

The brain tissue sections were precooled with heparinized physiological saline and 4% paraformaldehyde. The brain samples were collected, paraffin-embedded, and sectioned at 4 *μ*M coronally. Hematoxylin–eosin (H&E) staining was performed at the injury center by using an H&E staining kit (Solarbio, China) in accordance with the manufacturer's instructions.

### 2.5. Transmission Electron Microscopy

To observe morphological changes in the endoplasmic reticulum, the sections were stained with uranyl acetate and lead citrate for 15 min each. Then, the sections were observed under an H-7650 transmission electron microscope (Hitachi, Ibaraki, Japan) at an accelerating voltage of 80 kV. Regions exhibiting membrane alteration were photographed using a Gatan 830 CCD camera (Gatan, CA, USA).

### 2.6. Immunohistochemistry and TUNEL Staining

All sections were immersed in 4% paraformaldehyde for 20 min, washed three times with PBS (5 min/wash), immersed in 0.5% Triton for 20 min, and again washed with PBS (5 min/wash) three times. Then, the sections were first incubated with 10% goat serum for 30 min, followed by overnight incubation with primary antibodies of BCL-3 at 4°C. The sections were then washed three times with PBS and incubated with the secondary antibody at 37°C for 30 min. The slides were developed with DAB and observed under a bright-field microscope.

### 2.7. TUNEL

The TUNEL Detection Kit (Beyotime) was used for the TUNEL assay according to the manufacturer's instructions. In brief, the sections were treated with a red fluorescent-tagged enzyme solution and examined under a fluorescence microscope (DM2500; Leica Microsystems, Germany). Image-Pro Plus version 6.0 (Media Cybernetics, USA) was used to count TUNEL-positive cells emitting red fluorescence.

### 2.8. Real-Time Quantitative PCR (qPCR)

The expression of occludin and claudin-5 was evaluated *in vivo* (sham, ICH-24 h, and ICH-72 h) and *in vitro* (blank, ICH, ICH+NC, ICH+siBCL-3, ICH+vector, ICH+BCL-3, and ICH+siRNA BCL-3+2-C1HA). First, the total RNA was extracted using the TRIzol reagent (Invitrogen, USA). The primers were designed and synthesized by Sangon (Shanghai, China). The primers included the following: occludin forward primer (FR: 5′-CCCAGACCACTATGAAACCG-3′) and reverse primer (RP: 5′-CGCTCTCCTCTCTGTAGTCA-3′); claudin-5 FR (5′-GGTGTCTCAGAAGTACGAGC-3′) and RP (5′-TTCTTGTCGTAATCGCCGTT-3′); and GAPDH FR (5′-CCTCGTCTCATAGACAAGATGGT-3′) and RP (5′-GGGTAGAGTCATACTGGAACATG-3′). qPCR was conducted by using the HiScript II One Step qRT-PCR SYBR Green Kit (#Q221-01, Vazyme Biotech Co. Ltd., Nanjing, China). Finally, the expression levels of occludin and claudin-5 were tested on the ABI 7900 system (Foster City, CA, USA) and calculated by the 2^–*ΔΔ*Ct^ method, using GAPDH as the inner gene.

### 2.9. Western Blotting

Occludin and claudin-5 protein expressions were assessed through western blotting analysis. Proteins from cells and tissues were obtained through cell lysis with RIPA lysis buffer (#R0278, Sigma) and quantified using the BCA Protein Assay Kit (Beyotime, Jiangsu, China). The standard sodium dodecyl sulfate-polyacrylamide gel electrophoresis method was employed. Briefly, a 20 *μ*g protein was electrophoretically separated in a 10% sodium dodecyl sulfate separation gel under 100 V and a concentration gel under 120 V. The protein on the gel was transferred to a polyvinylidene fluoride (PVDF) membrane. The membrane was washed with 25 mL TBST (Tween-20: TBS = 1 : 1000) for 5 min and then blocked with 5% skimmed milk powder at 4°C overnight. Then, the samples were incubated for 1 h with primary antibody, rabbit anti-mouse polyclonal antibodies to BCL-3, occludin, claudin, p-PERK, ATF4, p-IRE1, GRP78, CHOP, and GAPDH followed by three washes with TBST (5 min/wash). The samples were then oscillated with the secondary antibody (HRP-labeled goat anti-rabbit IgG; 1 : 20000, BA1054, BOSTER Inc.) and incubated at room temperature for 40 min. The PVDF membrane was washed three times with 25 mL TBST (5 min/wash) and placed in the electrogenerated chemiluminescence (ECL) solution (ECL808-25, Biomiga, San Diego, CA) for 1 min to act fully. The excess liquid was removed, and the membrane was covered with a preservative film. The membrane was then photographed and observed using an X-ray machine (36209ES01, Shanghai Qcbio Science and Technologies Inc., Shanghai, China). The net density value of the band was calculated using Image-Pro Plus 6.0, with GAPDH as an internal reference band. All the experiments were repeated three times.

### 2.10. Enzyme-Linked Immunosorbent Assay

Inflammatory cytokines, including tumor necrosis factor-alpha (TNF-*α*) and interleukins (ILs, such as IL-1 and IL-6), were measured *in vivo* (sham, ICH-24 h, and ICH-72 h) and *in vitro* (blank, ICH, ICH+NC, ICH+siRNA BCL-3, ICH+vector, ICH+BCL-3, and ICH+siRNA BCL-3+2-CLHA). Before the rats were anesthetized, the blood was drawn and the serum was isolated. Serum IL-1, IL-6, and TNF-*α* levels were evaluated using enzyme-linked immunosorbent assay (ELISA) kits (#cat: 69-52313; #cat: 69-27187; #cat: 69-40254) obtained from MskBio (Wuhan, China).

### 2.11. RNA-seq and Data Processing

Total RNA in sham and ICH-72 h groups was extracted using TRIzol reagents (Invitrogen, USA). Samples with an OD_260/280_ of 1.8–2.0 and an RNA integrity number >7 were used for Illumina library preparation. Then, the libraries were constructed using TruSeq RNA Sample Prep Kits v2 (Illumina, USA). Finally, the cDNA libraries were sequenced on the Illumina NovaSeq 6000 platform with 2 × 150 bp pair-end reads in length.

Raw reads in the fastq format were processed using in-house Perl scripts. Clean reads were obtained by removing low-quality reads and reads with adapters or poly-N sequences. Q20, Q30, GC content, and sequence duplication level were calculated for the clean reads.

Cufflinks, HTSeq (v. 0.5.4 p3), and DESeq (v. 1.10.1) were used for assembly, mRNA expression level evaluation, and DEG identification, respectively. Genes with Log2|fold change (FC)| > 1 and *P* value < 0.05 were considered DEGs. The Gene Ontology (GO) analysis was performed using the DEGs.

### 2.12. Statistical Analysis

GraphPad Prism 8 (San Diego, CA, USA) was used for performing the statistical analysis. Unpaired Student's *t*-test and one-way ANOVA followed by post hoc (Tukey's multiple comparison analyses) were performed depending on the groups. Data are presented as mean ± standard deviation. *P* < 0.05 was considered to indicate a significant difference.

## 3. Results

### 3.1. ICH In Vivo Model Construction

**Figure 1:**
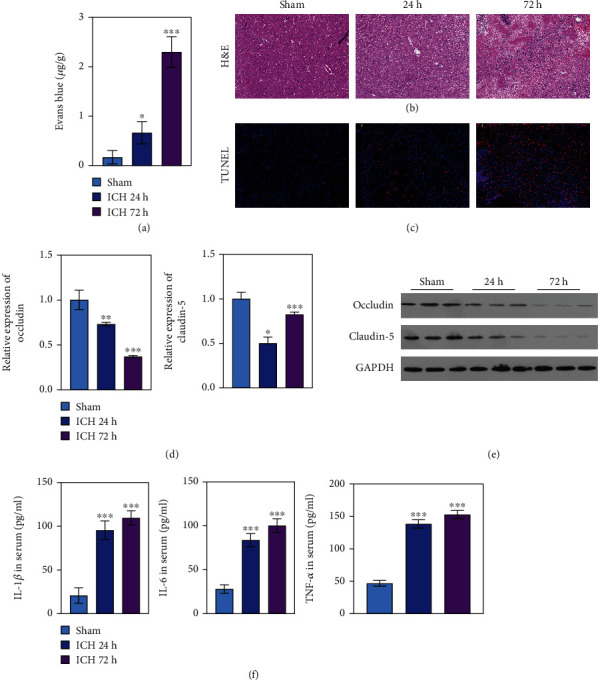
ICH *in vivo* model construction. (a) EBD extravasation in sham, ICH-24 h, and ICH-72 h. (b) H&E staining demonstrating the histopathology of sham, ICH-24 h, and ICH-72 h. (c) The tight junction proteins of occludin and claudin-5 as assessed by TUNEL staining. DAPI, blue; occludin and claudin-5, red. (d, e) qPCR and western blotting performed on sham, ICH-24 h, and ICH-72 h. (f) The inflammation factors of IL-1, IL-6, and TNF-*α* in the serum of sham, ICH-24 h, and ICH-72 h were evaluated.

The ICH model was constructed by injecting collagenase IV. Compared with the sham group, the EBD concentration was significantly increased in the ICH-72 h group, followed by the ICH-24 h group (*P* < 0.05; *P* < 0.001; Figure 1(a)). This indicates that the BBB was severely damaged in the ICH model, especially in the ICH-72 h group. H&E staining was used to assess histological changes in the rat brain. As shown in Figure 1(b), histopathology was normal, and no neuronal necrosis was observed in the sham group, whereas the neurons were disorderly arranged in the ICH-24 h and ICH-72 h groups. Expression levels of the tight junction proteins occludin and claudin-5 in the brain tissues were evaluated through qPCR and western blotting. As shown in Figure 1(c), TUNEL assay results revealed that apoptosis was increased in the ICH-24 h and ICH-72 h groups compared with the sham group (Figure 1(c)**)**. qPCR and western blotting analyses revealed that occludin and claudin-5 expression was significantly lower in the ICH-24 h and ICH-72 h groups than in the sham group (*P* < 0.05; *P* < 0.001; Figures 1(d) and 1(e)), which revealed an increased BBB permeability in ICH.

Then, we assessed serum levels of the inflammatory factors IL-1*β*, IL-6, and TNF-*α* in the three groups. IL-1, IL-6, and TNF-*α* levels significantly increased in the ICH models with time compared with the sham group (*P* < 0.001; Figure 1(f)). These results confirmed that the BBB was severely damaged, and the levels of the inflammatory factors increased in the ICH models, indicating a successfully constructed ICH rat model.

### 3.2. RNA-seq Technology for Biomarker Identification

**Figure 2:**
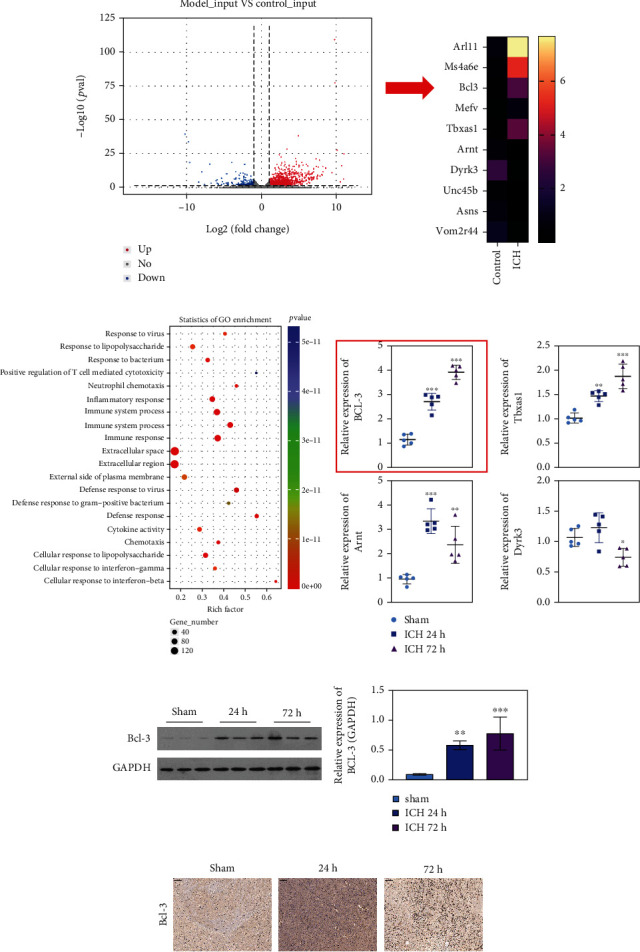
RNA-seq technology on biomarker identification. (a) The volcano plot of DEGs in sham and the ICH group. (b) The top 10 dysregulated DEGs in comparisons. (c) The GO analysis of DEGs in comparisons. (d) The qPCR analysis in sham, ICH-24 h, and ICH-72 h. (e, f) Western blotting and IF analyses of BCL-3 in sham, ICH-24 h, and ICH-72 h. ^∗∗^*P* < 0.01; ^∗∗∗^*P* < 0.001.

RNA-seq was performed to identify DEGs, showing as the volcano plot (Figure 2(a)). The DEGs of Arl11, Ms4a6e, BCL-3, Mefv, Tbxas1, Arnt, Dyrk3, Unc45b, Asns, and Vom2r44 were identified as the top 10 dysregulated DEGs in the ICH group compared with the control group (Figure 2(b)). The DEGs were mainly enriched in the GO in terms of the inflammatory response, immune system process, and cytokine activity (Figure 2(c)). Then, BCL-3, Tbxas1, Arnt, and Dyrk3 expression in the sham, ICH-24 h, and ICH-72 h samples were evaluated. Compared with the sham group, BCL-3 and Tbxas1 were mostly upregulated in the ICH-72 h group, followed by the ICH-24 h group (*P* < 0.01, *P* < 0.001; Figure 2(d)). BCL-3 exhibited a higher fold change than Tbxas1. Then, BCL-3 expression in the sham, ICH-24 h, and ICH-72 h groups was investigated through western blotting. BCL-3 expression was the highest in the ICH-72 h group, followed by the ICH-24 h group (*P* < 0.01, *P* < 0.001; Figures 2(e) and 2(f)). The western blotting and IHC analyses exhibited similar results as qPCR. Taken together, BCL-3 was identified as a biomarker of ICH. However, verification experiments are warranted.

### 3.3. BCL-3 Promotes ICH Progression by Increasing BBB Permeability, Endoplasmic Reticulum Stress, Inflammation, and Cell Apoptosis

**Figure 3:**
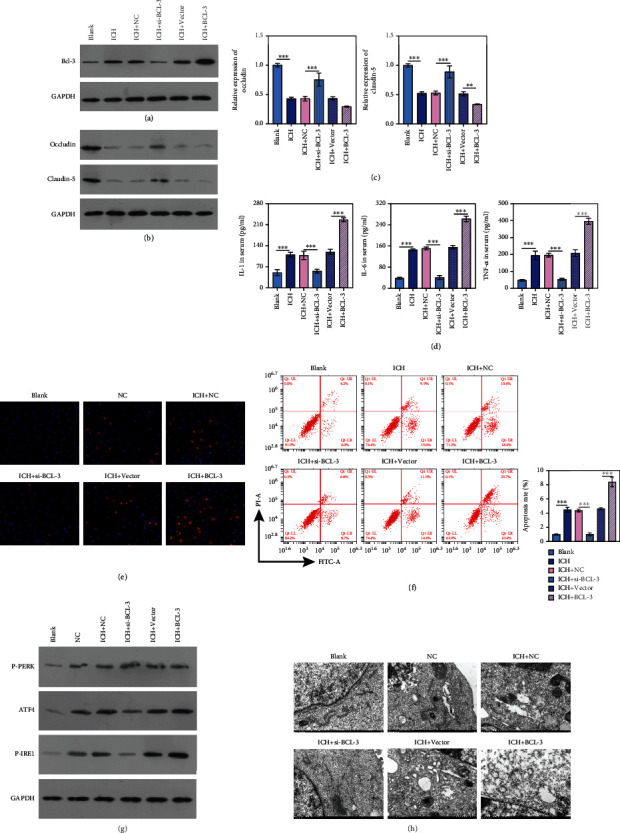
BCL-3 promotes ICH progression by increasing BBB permeability, ERS, inflammation, and cell apoptosis. (a, b) The expression level of BCL-3, occludin, and claudin-5 was tested by western blotting. (c) The expression level of occludin and claudin-5 evaluated by qPCR. (d) ELISA measured the levels of inflammatory factors of IL-6, IL-1, and TNF-*α*. (e, f) TUNEL and FCM analyses were performed to measure cell apoptosis. (g) Western blotting performed on ERS-related proteins, including p-PERK, ATF4, and p-IRE1*α*. (h) TEM observation on the endoplasmic reticulum. ^∗∗∗^*P* < 0.001.

The BCL-3 expression level was significantly downregulated in ICH+siRNA BCL-3 and upregulated in ICH+BCL-3 compared with the ICH group (Figure 3(a)). The tight junction proteins occludin and claudin-5 were significantly upregulated in ICH+siBCL-3 and downregulated in ICH+BCL-3, revealing an increased BBB permeability with BCL-3 overexpression (*P* < 0.01; *P* < 0.001; Figures 3(a)–3(c)). IL-6, IL-1*β*, and TNF-*α* levels were significantly decreased in ICH+siBCL-3 compared with ICH+NC and increased in ICH+BCL-3 compared with ICH+vector (*P* < 0.001; Figure 3(d)), demonstrating a proinflammatory role of BCL-3 in ICH. Then, we evaluated the role of BCL-3 in the regulation of cell apoptosis in ICH.

For this purpose, the TUNEL assay and FCM were performed. As shown in Figures 3(e) and 3(f), cell apoptosis increased in the ICH group compared with the blank, and BCL-3 overexpression significantly enhanced cell apoptosis compared with the ICH group (Figures 3(e) and 3(f)).

To explore whether BCL-3 regulation is related to endoplasmic reticulum stress (ERS) in ICH, three signal pathways related to the unfolded protein response (UPR) in ERS, namely, p-PERK, ATF4, and p-IRE1*α*, were detected and analyzed through western blotting. Compared with the blank group, p-IRE1*α* was significantly upregulated in the ICH group, which was significantly increased with BCL-3 overexpression and downregulated with BCL-3 silencing. This indicated that ERS was elevated with BCL-3 overexpression through the IRE1*α* signaling pathway. Expression of the other proteins, p-PERK and ATF4, exhibited no significant differences in the groups. Therefore, p-IRE1*α* was selected for the subsequent experiments **(**Figure 3(g)). As observed through transmission electron microscopy (TEM), the endoplasmic reticulum was reduced and deformed in the ICH group, especially in ICH+BCL-3. This effect was reversed with BCL-3 silencing (Figure 3(h)).

### 3.4. Silencing BCL-3 Slows Down ICH Progression by Reducing BBB Permeability and Inflammation and Terminating Cell Apoptosis through ERS

**Figure 4:**
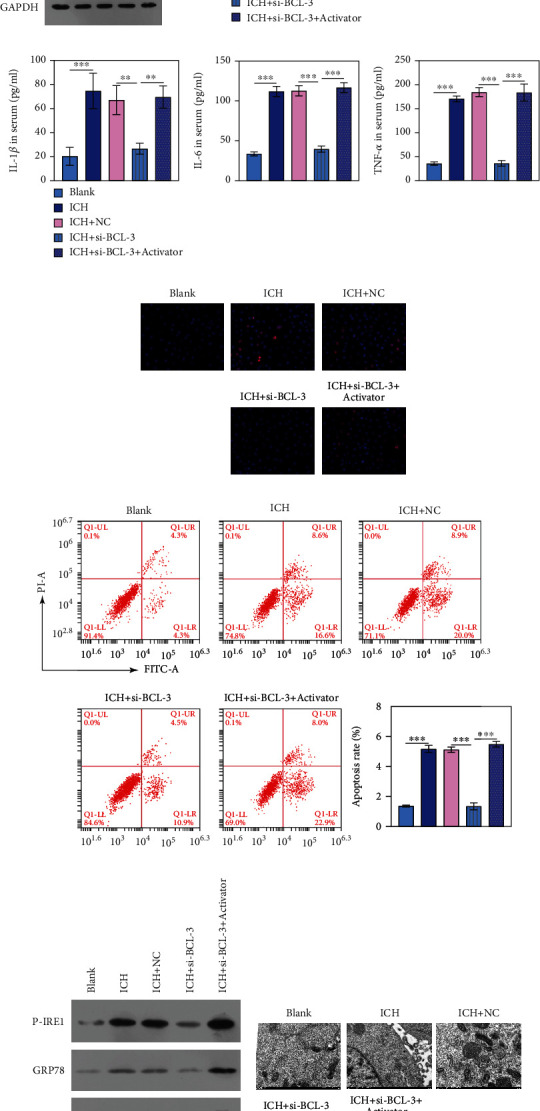
ERS activator reversed the role of BCL-3 siRNA on BBB permeability and inflammation and terminated cell apoptosis in BMECs. (a) The expression level of BCL-3 was tested by western blotting. (b) The expression level of occludin and claudin-5 was evaluated by qPCR. (c) ELISA measured the levels of inflammatory factors of IL-6, IL-1*β*, and TNF-*α*. (d, e) TUNEL and FCM were applied to evaluate cell apoptosis. (f) Western blotting performed on ERS-related proteins, including p-IRE1*α*, GRP78, and CHOP. (f) TEM was performed to observe ER. ^∗∗∗^*P* < 0.001.

To further investigate the role of ERS in regulating BBB permeability, inflammation, and cell apoptosis in ICH and 2-CLHA, an ERS activator was used in the ICH *in vitro* model transfected with siRNA 2-CLHA had no effect on BCL-3 expression (Figure 4(a)). The expression analyses performed through qPCR revealed that the ERS activator reversed the BCL-3 siRNA-mediated expression enhancement of occludin and claudin-5 (Figure 4(b)). Furthermore, IL-1*β*, IL-6, and TNF-*α* suppressed by siRNA BCL-3 were rescued by the ERS activator (Figure 4(c)). As shown in Figures 4(d) and 4(e), the ERS activator reversed the protective effect of siRNA BCL-3 on cell apoptosis in the ICH group (Figures 4(d) and 4(e)).

GRP78 is a key regulator of ERS, and CHOP is a proapoptosis biomarker of ERS. Therefore, GRP78 and CHOP expressions can indicate ERS progression. p-IRE1*α*, GRP78, and CHOP protein expressions were significantly upregulated in ICH+siBCL-3+2-CLHA compared with ICH+siBCL-3 (Figure 4(f)). TEM revealed that the released endoplasmic reticulum by ICH+BCL-3 siRNA can be reversed by ICH+BCL-3 siRNA+2-CLHA (Figure 4(g)).

### 3.5. Silencing BCL-3 Reversed ICH by Promoting BBB Permeability and ERS and Reducing Inflammation In Vivo

**Figure 5:**
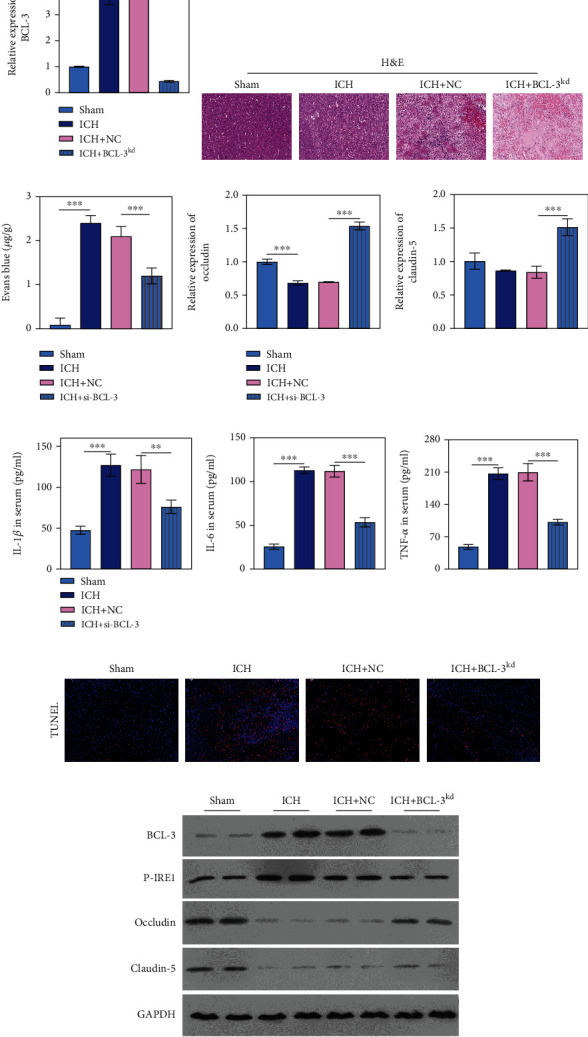
Effect of BCL-3 knockdown on BBB permeability and ERS, reducing inflammation *in vivo*. (a) qRT-PCR was performed to measure the BCL-3 expression on the brain. (b) H&E staining demonstrating the histopathology of the brain. (c) BBB permeability was evaluated by Evans blue staining. (d) The tight junction proteins of occludin and claudin-5 measured by qRT-PCR. (e) The inflammation factors of IL-1, IL-6, and TNF-*α* in the serum. (f) Apoptosis was evaluated by TUNEL. (g) ERS-related proteins were measured by western blotting.

We further verified the role of BCL-3 in ICH progression *in vivo*. BCL-3 expression was elevated in ICH and was suppressed by BCL-3 knockdown (Figure 5(a)). H&E staining displayed that histopathologically, the cells were gradually disorganized, and neuronal necrosis was observed in ICH. BCL-3 knockdown exerted a protective role in the brain (Figure 5(b)). As shown in Figure 5(c), BBB permeability increased in animals from the ICH group, whereas this effect was reversed by BCL-3 knockdown (Figure 5(c)), which was accompanied by elevated occludin and claudin-5 expression **(**Figure 5(d)**)**. Levels of the inflammatory factors IL-1*β*, IL-6, and TNF-*α* were significantly decreased in ICH+BCL-3^kd^ compared with ICH+NC (*P* < 0.001; Figure 5(e)). The TUNEL assay demonstrated that apoptosis was increased in the brain of rats from the ICH group, and apoptosis was reversed with siRNA BCL-3 (Figure 5(f)). The p-IRE1 expression level was downregulated in ICH+BCL-3^kd^, as observed through western blotting (Figure 5(g)).

## 4. Discussion

High mortality and morbidity related to ICH have made the screening of biomarkers for ICH extremely critical. Numerous studies have recently been conducted to identify biomarkers and investigate the molecular mechanisms underlying ICH. For example, Gareev et al. [15] demonstrated that some miRNAs (namely, miR-181b, miR-223, miR-155, and miR-145) might act as a potential noninvasive tool for ICH detection. Zhang et al. [16] identified the CYP4F2 protein as a potential biomarker of ICH for prevention and treatment assessment. The molecular methods allowed for ICH diagnosis, prevention, and treatment assessment. Our present study elucidated the role of BCL-3 in ICH for the first time. RNA-seq was employed for biomarker identification, and BCL-3 was identified as a DEG dysregulated in the ICH rats compared with the control rats. Then, the role of BCL-3 was verified *in vitro* and *in vivo*. BCL-3 overexpression was found to promote ICH progression by increasing BBB permeability, ERS, inflammation, and cell apoptosis. Interfering with BCL-3 can alleviate ERS, apoptosis, and inflammatory responses.

BCL-3 is a member of the I*κ*B family, which regulates gene transcription as a nuclear cofactor [17]. P38 MAPK could upregulate NF-*κ*B and p53, thereby promoting I*κ*B phosphorylation and dissociating NF-*κ*B and I*κ*B complexes [18, 19]. NF-*κ*B activation translocates BCL-3 into the nucleus, where it regulates the transcription of proinflammatory cytokine genes such as TNF-*α*, IL-1*β*, and IL-6 [20–22]. BCL-3 has been reported as a biomarker for several diseases. For example, BCL-3 is a sensitive marker for renal damage in several diseases [23, 24]. High BCL-3 expression can promote resistance to alkylating chemotherapy in gliomas [25]. In cancer, BCL-3 limits the impact of the enabling hallmarks of cell plasticity and tumor-promoting inflammation [26]. However, the role of BCL-3 in ICH has rarely been investigated. Our present study found that BCL-3 was significantly upregulated in ICH rats. Then, the role of BCL-3 in ICH was evaluated in terms of BBB permeability, inflammation, cell apoptosis, and ERS.

The disruption of the BBB, maintained by tight junctions between adjacent brain microvascular endothelial cells, induces edema, thus causing ICH-induced brain injury [27]. In addition, alterations in the levels of tight junction proteins may contribute to increased BBB permeability, thereby aggravating BBB dysfunction [28]. We found that BCL-3 can promote ICH progression by increasing BBB permeability.

The endoplasmic reticulum is a vital organelle for protein synthesis, folding, and secretion in eukaryotic cells [29]. Cells have evolved a complete set of mechanisms for supervising and assisting protein folding and modification within the endoplasmic reticulum. When misfolded proteins accumulate, cells respond to them through a series of signal transduction pathways.

The endoplasmic reticulum has a strong homeostatic system to realize the stability of its function. However, destructive factors induce homeostasis of endoplasmic reticulum function, resulting in ERS. If endoplasmic reticulum dysfunction persists, cells will eventually initiate caspase-12-dependent apoptosis [30]. These responses are collectively called the UPR. Cells under ERS can regulate the expression/activation of ERS-related proapoptotic molecules, such as CHOP and caspase-12, and prosurvival molecules, such as GADD34 and BiP, which ultimately determine whether cells adapt or undergo apoptosis [31, 32].

BCL-3 promotes ICH by promoting inflammation. Caglayan et al. [33] demonstrated that BCL-3 acts as an inflammatory factor increased by HgCl_2_, indicating the anti-inflammatory effect of HgCl_2_ on liver injury. Legge et al. [26] also exhibited that BCL-3 prevents inflammation, which might be effective in treating inflammatory diseases. However, the role of BCL-3 in our study was varied. BCL-3 overexpression increased the levels of inflammatory factors, including IL-1, IL-6, and TNF-*α*. Moreover, the GO analysis in this study also demonstrated the inflammatory response of DEGs in ICH. Therefore, BCL-3 exhibited a proinflammatory role in ICH progression.

## 5. Conclusion

We conclude that BCL-3 was overexpressed in ICH rats and promoted ICH progression by increasing BBB permeability, inflammation, and cell apoptosis through ERS.

## Data Availability

All data are available from the corresponding author with reasonable request.

## References

[1] Joseph M. J., Caliaperumal J., Schlichter L. C. (2016). After intracerebral hemorrhage, oligodendrocyte precursors proliferate and differentiate inside white-matter tracts in the rat striatum. *Translational stroke research*.

[2] Behrouz R. (2016). Re-exploring tumor necrosis factor alpha as a target for therapy in intracerebral hemorrhage. *Translational stroke research*.

[3] Fei X., He Y., Chen J. (2019). The role of toll-like receptor 4 in apoptosis of brain tissue after induction of intracerebral hemorrhage. *Journal of Neuroinflammation*.

[4] Dang G., Yang Y., Wu G., Hua Y., Keep R. F., Xi G. (2017). Early erythrolysis in the hematoma after experimental intracerebral hemorrhage. *Translational stroke research*.

[5] Sukumari-Ramesh S., Alleyne C. H., Dhandapani K. M. (2016). The histone deacetylase inhibitor suberoylanilide hydroxamic acid (SAHA) confers acute neuroprotection after intracerebral hemorrhage in mice. *Translational stroke research*.

[6] Aronowski J., Zhao X. (2011). Molecular pathophysiology of cerebral hemorrhage: secondary brain injury. *Stroke*.

[7] Zhang Z., Song Y., Li F., Xu Z., Huang Q. (2020). Inhibiting nuclear factor-*κ*B at different stages after intracerebral hemorrhage can influence the hemorrhage-induced brain injury in experimental models in vivo. *Brain Research Bulletin*.

[8] Shen H., Liu C., Zhang D. (2017). Role for RIP1 in mediating necroptosis in experimental intracerebral hemorrhage model both _in vivo_ and _in vitro_. *Cell death disease*.

[9] Sultan W., Machado L. G. D. D., Ali M. G. (2022). MicroRNAs as biomarkers in spontaneous intracerebral hemorrhage: a systematic review of recent clinical evidence. *Clinical Neurology Neurosurgery*.

[10] Yang C., Wu J., Lu X., Xiong S., Xu X. (2022). Identification of novel biomarkers for intracerebral hemorrhage via long noncoding RNA-associated competing endogenous RNA network. *Molecular Omics*.

[11] Cheng X., Ander B. P., Jickling G. C. (2020). MicroRNA and their target mRNAs change expression in whole blood of patients after intracerebral hemorrhage. *Journal of Cerebral Blood Flow Metabolism*.

[12] Walsh K. B., Zhang X., Zhu X. (2019). Intracerebral hemorrhage induces inflammatory gene expression in peripheral blood: global transcriptional profiling in intracerebral hemorrhage patients. *DNA and Cell Biology*.

[13] Kung T. F., Wilkinson C. M., Dirks C. A., Jickling G. C., Colbourne F. (2021). Glibenclamide does not improve outcome following severe collagenase-induced intracerebral hemorrhage in rats. *PLoS One*.

[14] Guo Q., Xie M., Guo M., Yan F., Li L., Liu R. (2021). ZEB2, interacting with MDM2, contributes to the dysfuntion of brain microvascular endothelial cells and brain injury after intracerebral hemorrhage. *Cell Cycle*.

[15] Gareev I., Yang G., Sun J. (2020). Circulating microRNAs as potential noninvasive biomarkers of spontaneous intracerebral hemorrhage. *World Neurosurgery*.

[16] Zhang J., Su X., Qi A. (2021). Metabolomic profiling of fatty acid biomarkers for intracerebral hemorrhage stroke. *Talanta*.

[17] Ohno H., Takimoto G., McKeithan T. W. (1990). The candidate proto-oncogene _bcl_ -3 is related to genes implicated in cell lineage determination and cell cycle control. *Cell*.

[18] Yang D., Tan X., Lv Z. (2016). Regulation of Sirt1/Nrf2/TNF-*α* signaling pathway by luteolin is critical to attenuate acute mercuric chloride exposure induced hepatotoxicity. *Scientific Reports*.

[19] Saccani S., Pantano S., Natoli G. (2002). p38-dependent marking of inflammatory genes for increased NF-*κ*B recruitment. *Nature Immunology*.

[20] Caglayan C., Temel Y., Kandemir F. M., Yildirim S., Kucukler S. (2018). Naringin protects against cyclophosphamide-induced hepatotoxicity and nephrotoxicity through modulation of oxidative stress, inflammation, apoptosis, autophagy, and DNA damage. *Environmental Science Pollution Research*.

[21] Liu B., Yu H., Baiyun R. (2018). Protective effects of dietary luteolin against mercuric chloride-induced lung injury in mice: involvement of AKT/Nrf2 and NF-*κ*B pathways. *Food Chemical Toxicology*.

[22] Turk E., Kandemir F. M., Yildirim S., Caglayan C., Kucukler S., Kuzu M. (2019). Protective effect of hesperidin on sodium arsenite-induced nephrotoxicity and hepatotoxicity in rats. *Biological trace element research*.

[23] Chen R., Wang L., Liu S. (2017). Bcl-3 is a novel biomarker of renal fibrosis in chronic kidney disease. *Oncotarget*.

[24] Nakayama H., Echizen H., Gomi T. (2009). Urinary lipocalin-type prostaglandin D synthase: a potential marker for early gentamicin-induced renal damage?. *Therapeutic drug monitoring*.

[25] Wu L., Bernal G. M., Cahill K. E. (2018). BCL3 expression promotes resistance to alkylating chemotherapy in gliomas. *Science translational medicine*.

[26] Legge D. N., Chambers A. C., Parker C. T., Timms P., Collard T. J., Williams A. C. (2020). The role of B-cell lymphoma-3 (BCL-3) in enabling the hallmarks of cancer: implications for the treatment of colorectal carcinogenesis. *Carcinogenesis*.

[27] Xing G., Zhao T., Zhang X. (2020). Astrocytic sonic hedgehog alleviates intracerebral hemorrhagic brain injury via modulation of blood-brain barrier integrity. *Frontiers in Cellular Neuroscience*.

[28] Zlokovic B. V. (2008). The blood-brain barrier in health and chronic neurodegenerative disorders. *Neuron*.

[29] Wang M., Kaufman R. J. (2016). Protein misfolding in the endoplasmic reticulum as a conduit to human disease. *Nature*.

[30] Zhu W., Zhang R., Liu S. (2021). The effect of nanoparticles of cobalt–chromium on human aortic endothelial cells in vitro. *Journal of Applied Toxicology*.

[31] Marciniak S. J., Yun C. Y., Oyadomari S. (2004). CHOP induces death by promoting protein synthesis and oxidation in the stressed endoplasmic reticulum. *Genes development*.

[32] Iurlaro R., Muñoz-Pinedo C. (2016). Cell death induced by endoplasmic reticulum stress. *The Febs Journal*.

[33] Caglayan C., Kandemir F. M., Darendelioğlu E., Yıldırım S., Kucukler S., Dortbudak M. B. (2019). Rutin ameliorates mercuric chloride-induced hepatotoxicity in rats via interfering with oxidative stress, inflammation and apoptosis. *Journal of Trace Elements in Medicine Biology*.

